# Combinatorial Guidance by CCR7 Ligands for T Lymphocytes Migration in Co-Existing Chemokine Fields

**DOI:** 10.1371/journal.pone.0018183

**Published:** 2011-03-25

**Authors:** Saravanan Nandagopal, Dan Wu, Francis Lin

**Affiliations:** 1 Department of Physics and Astronomy, University of Manitoba, Winnipeg, Manitoba, Canada; 2 Department of Biosystems Engineering, University of Manitoba, Winnipeg, Manitoba, Canada; 3 Department of Biological Sciences, University of Manitoba, Winnipeg, Manitoba, Canada; 4 Department of Immunology, University of Manitoba, Winnipeg, Manitoba, Canada; New York University, United States of America

## Abstract

Chemokines mediate the trafficking and positioning of lymphocytes in lymphoid tissues that is crucial for immune surveillance and immune responses. In particular, a CCR7 ligand, CCL21, plays important roles in recruiting T cells to secondary lymphoid tissues (SLT). Furthermore, CCL21 together with another CCR7 ligand, CCL19, direct the navigation and compartmentation of T cells within SLT. However, the distinct roles of these two chemokines for regulating cell trafficking and positioning are not clear. In this study, we explore the effect of co-existing CCL19 and CCL21 concentration fields on guiding T cell migration. Using microfluidic devices that can configure single and superimposed chemokine fields we show that under physiological gradient conditions, human peripheral blood T cells chemotax to CCL21 but not CCL19. Furthermore, T cells migrate away from the CCL19 gradient in a uniform background of CCL21. This repulsive migratory response is predicted by mathematical modeling based on the competition of CCL19 and CCL21 for CCR7 signaling and the differential ability of the two chemokines for desensitizing CCR7. These results suggest a new combinatorial guiding mechanism by CCL19 and CCL21 for the migration and trafficking of CCR7 expressing leukocytes.

## Introduction

Migratory responses of cells to cellular guiding signals play important roles in regulating a wide range of physiological and pathological processes such as inflammation and autoimmune diseases, wound healing, neuron guidance, embryogenesis, and cancer metastasis [1], [2], [3], . In particular, chemoattractant gradients guide the migration of immune cells (i.e. chemotaxis), orchestrating cell trafficking and positioning in tissues [7], [8]. It has been shown that leukocytes express multiple different chemoattractant receptors in a cell subset dependent manner, and can integrate multiple co-existing chemotactic signals to direct their migration to specific targets in tissues that enable immune surveillance and immune responses [9], [10]. Such a multiple chemoattractants-based guiding mechanism relies on chemotactic signaling transduction through chemoattractant and their different specific cell surface receptors. In contrast, some chemoattractants share a common receptor for triggering chemotactic signaling such as chemokines CCL19 and CCL21 and their shared receptor CCR7 expressed in lymphocytes subsets and dendritic cells (DCs) [11], [12], [13], [14]. However, the mechanism of multiple chemoattractants with a common cell receptor for guiding cell migration is unclear.

Chemokine receptor CCR7 and its two ligands, chemokine CCL19 and CCL21, are important players in regulating lymphocytes and DCs trafficking in secondary lymphoid tissues (SLT) such as lymph nodes (LNs) [11], [12], [13], [14]. CCL19 and CCL21 are co-expressed in LNs with different expression patterns. CCL19 is only produced and presented in T cell zone (TCZ) in humans and mouse LNs [11], [13], [15], [16]. In contrast, CCL21 is produced in TCZ and is transcytosed to high endothelial venules (HEV) in human [13], [17], [18], and is produced and presented in both TCZ and HEV in mouse LNs [19], [20]. Inside TCZ, it has been shown that CCL19 and CCL21 are co-expressed by reticular cells with more CCL21-expressing cells than CCL19-expressing cells in the periphery of TCZ [21], suggesting the size of the CCL21 producing tissue in TCZ is possibly larger than the CCL19 producing tissue. Therefore, the profiles of overlapping CCL19 and CCL21 fields can be different in different sub-regions of TCZ. Furthermore, the production levels of CCL19 and CCL21 in SLT are significantly different with up to 100-fold higher of CCL21 production than CCL19 [15], [19] but the exact difference is not defined in sub-regions. In addition, CCL19 only exhibits soluble patterns in SLT whereas CCL21 is found in both soluble and immobilized forms [22]. At the cellular level, CCL19 and CCL21 have similar binding affinity with CCR7 and they are similar in inducing calcium immobilization and G protein activation [23]. However, only CCL19 but not CCL21 robustly desensitizes and internalizes CCR7 [14], [16], [24]. Although both CCL19 and CCL21 are potent chemoattractants for T cells as shown using *in-vitro* chemotaxis assays, their distinct roles in regulating T cell trafficking in SLT remain unclear. It has been shown that CCL21 but not CCL19 is required for T cells and DCs recruitment to SLT using CCL19/21 deficient mice and CCL19 deficient mice [20], [25], [26]. This finding together with the significantly lower production level of CCL19 further complicates the role of CCL19 in lymphocytes and DCs trafficking in SLT. Altogether, the differential expression patterns of CCL19 and CCL21 in SLT and their differential ability for desensitizing CCR7 and for recruiting T cells and DCs to SLT present a complex and unclear picture of CCR7 ligands guided T cell migration and trafficking in SLT.

In the present study, we hypothesize that the different profiles of co-existing CCL19 and CCL21 fields in sub-regions of LNs together with the differential ability of CCL19 and CCL21 for desensitizing CCR7 provide a mechanism for fine tuning T cell trafficking in LNs. We employed a microfluidics-based approach to quantitatively analyze T cell migration in-vitro in different configurations of co-existing CCL19 and CCL21 fields that mimic the physiological conditions in different regions of LNs and the results are explained by mathematical modeling and computer simulations. The experimental and modeling results allow us to formulate a possible combinatorial guiding mechanism by co-existing CCL19 and CCL21 gradient fields for T cell migration and trafficking.

## Results and Discussion

### Physiological CCL21 but not CCL19 gradient attracts T cells

**Figure 1:**
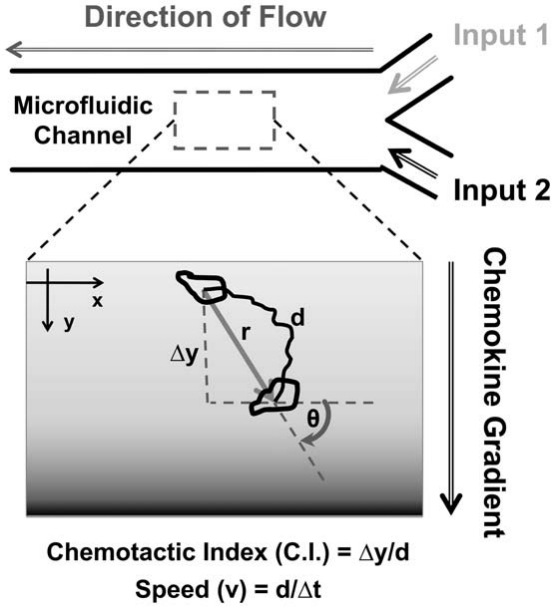
Illustration of cell migration experiments using microfluidic devices and data analysis methods.

**Figure 2:**
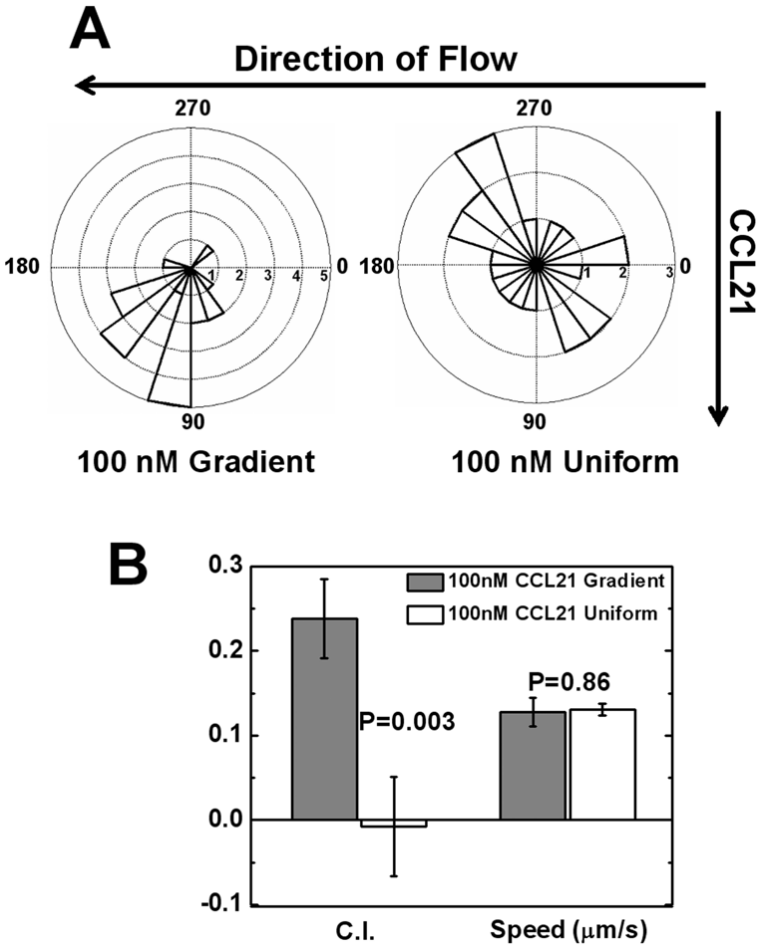
T cell migration in a gradient or a uniform field of CCL21. (**A**) Angular histograms show T cells orient randomly in a 100 nM uniform CCL21 field, but toward a 100 nM CCL21 gradient (**B**) Comparison of chemotactic index (C.I.) and speed of cells in a 100 nM uniform CCL21 field or a 100 nM CCL21 gradient show random migration in the 100 nM uniform CCL21 field, but chemotaxis in the 100 nM CCL21 gradient with similar speed. The error bars represent the standard error of the mean (s.e.m.). The *p* values for each comparison from 2-sample *t* test are shown. Positive C.I. indicates cells migrate toward the gradients.

**Figure 3:**
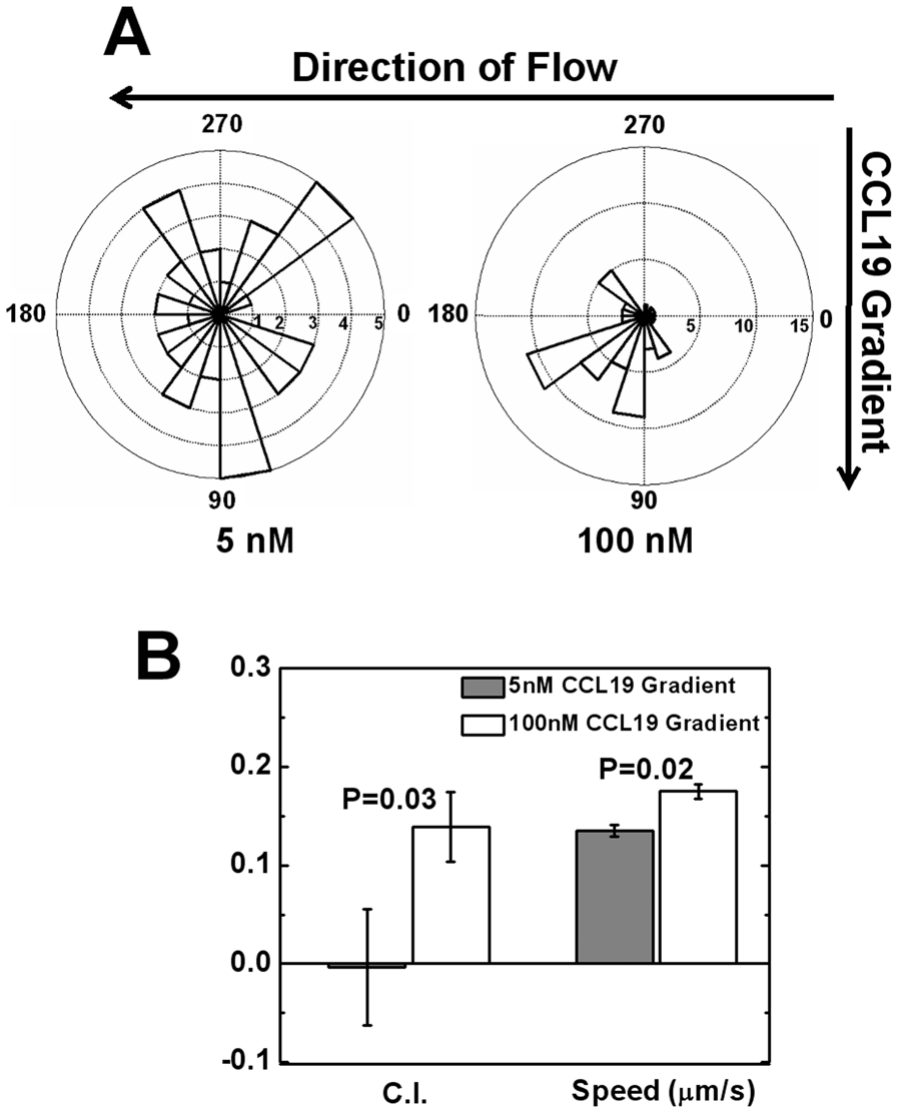
T cell migration in CCL19 gradients. (**A**) Angular histograms show T cells orient randomly in a 5 nM CCL19 gradient, but toward a 100 nM CCL19 gradient (**B**) Comparison of chemotactic index (C.I.) and speed of cells in a 5 nM CCL19 gradient or a 100 nM CCL19 gradient show random migration in the 5 nM CCL19 gradient, but chemotaxis in the 100 nM CCL19 gradient with higher speed in the 100 nM CCL19 gradient. The error bars represent the standard error of the mean (s.e.m.) The *p* values for each comparison from 2-sample *t* test are shown. Positive C.I. indicates cells migrate toward the gradients.

As illustrated in Figure 1, we employed a microfluidic device that can precisely configure stable single or superimposed chemokine gradients by controlled mixing of continuous flows inside a microfluidic channel for quantitative cell migration analysis, and we used activated human peripheral blood T cells as a model cell system. In the first, we tested the migration of T cells in a CCL19 or a CCL21 concentration field with physiological doses (i.e. 100 nM for CCL21 and 5 nM for CCL19, which were selected based on *in-vivo* studies of CCL19 and CCL21 expression [19] and the saturation chemokine concentration for T cell chemotaxis *in-vitro* [12], [27]. Our results show that T cells strongly chemotax to the 100 nM CCL21 gradient (Figure 2 and Video S1). A high percentage of cells migrate toward the gradient with a high chemotactic index. In the uniform field of 100 nM CCL21, T cells migrate randomly (Figure 2 and Video S2). The speed of cells is similar in the gradient and uniform CCL21 field. These results confirm CCL21 as a potent chemoattractant for T cells and suggest its role in T cell recruitment to TCZ. In contrast, T cells migrate randomly in a 5 nM CCL19 gradient (Figure 3 and Video S3). In a super-physiological 100 nM CCL19 gradient, T cells show strong chemotaxis (Figure 3 and Video S4). Interestingly, T cells maintain similar migration speeds in the 5 nM CCL19 gradient when compared with those observed in a 100 nM CCL21 gradient or 100 nM uniform CCL21 field (Figure 2), suggesting the motile nature of activated T cells. These results confirm that CCL19 can act as a chemoattractant for T cells at a super-physiological concentration. However, the much lower physiological dose of a CCL19 gradient is not sufficient to attract T cells. To further validate the 100 nM CCL21 gradient as a chemoattractant for T cells in SLT, we tested the condition of competing gradients of 100 nM CCL21 and 5 nM CCL19. Our results show 60% of cells migrate toward the 100 nM gradient, suggesting that the 100 nM CCL21 gradient attracts cells even in the presence of an opposing 5 nM CCL19 gradient. Interestingly we found the chemotactic index (C.I.) toward the CCL21 gradient is significantly reduced (i.e. 0.04±0.04) comparing to it in the single CCL21 gradient (i.e. 0.24±0.04). We speculate that although the opposing 5 nM CCL19 gradient does not attract cells by itself, it may still have an effect on cell chemotaxis to the 100 nM CCL21 gradient at the quantitative level. While it is interesting, this aspect of the study is beyond the focus of the current paper and thus is not discussed further. Collectively, the results from the cell migration experiments in single and competing CCL19 and CCL21 fields indicate that at physiological concentrations, CCL21 but not CCL19 serves as a chemoattractant for T cell migration, which is consistent with previous *in-vivo* studies showing CCL21 alone is sufficient for T cells and DCs recruitment to SLT [20], [25], [26].

### T cells migrate randomly in superimposed CCL19 and CCL21 uniform fields at physiological concentrations

**Figure 4:**
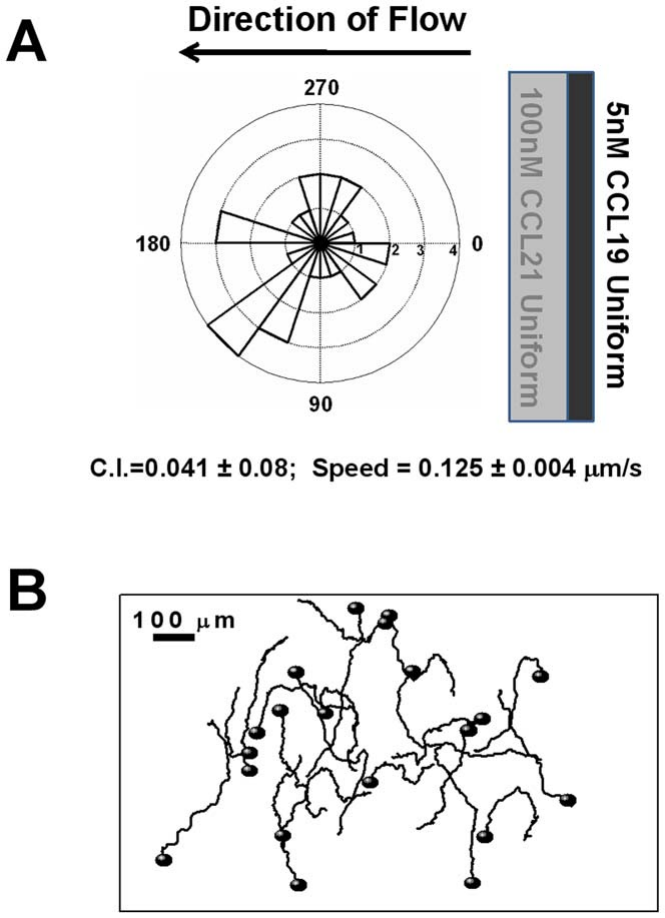
T cell migration in “double-uniform” CCL19 and CCL21 fields. (**A**) Angular histogram shows random orientation of T cells in superimposed 5 nM CCL19 and 100 nM CCL21 uniform fields. Chemotactic index (C.I.) and the speed of cells are shown with the errors represented as the standard error of the mean (s.e.m.) Positive C.I. indicates cells migrate toward the gradients. (**B**) Selected cell tracks from a representative experiment showing cells migrate randomly.

Both CCL19 and CCL21 are produced in TCZ, wherein uniform concentration fields of both chemokines are expected. Thus, we tested T cell migration in superimposed CCL19 and CCL21 uniform fields at physiological concentrations (i.e. 100 nM for CCL21 and 5 nM for CCL19) using microfluidic devices. Our results show that T cells exhibit random orientation and migration in the “double uniform” chemokine fields (Figure 4 and Video S5). However, the speed of T cells is similar to it in single CCL19 or CCL21 gradient or uniform fields at the physiological concentrations. Thus, CCL19 does not necessarily enhance T cell motility in TCZ.

### T cells migrate away from the physiological CCL19 gradient in the presence of a uniform background of 100 nM CCL21

**Figure 5:**
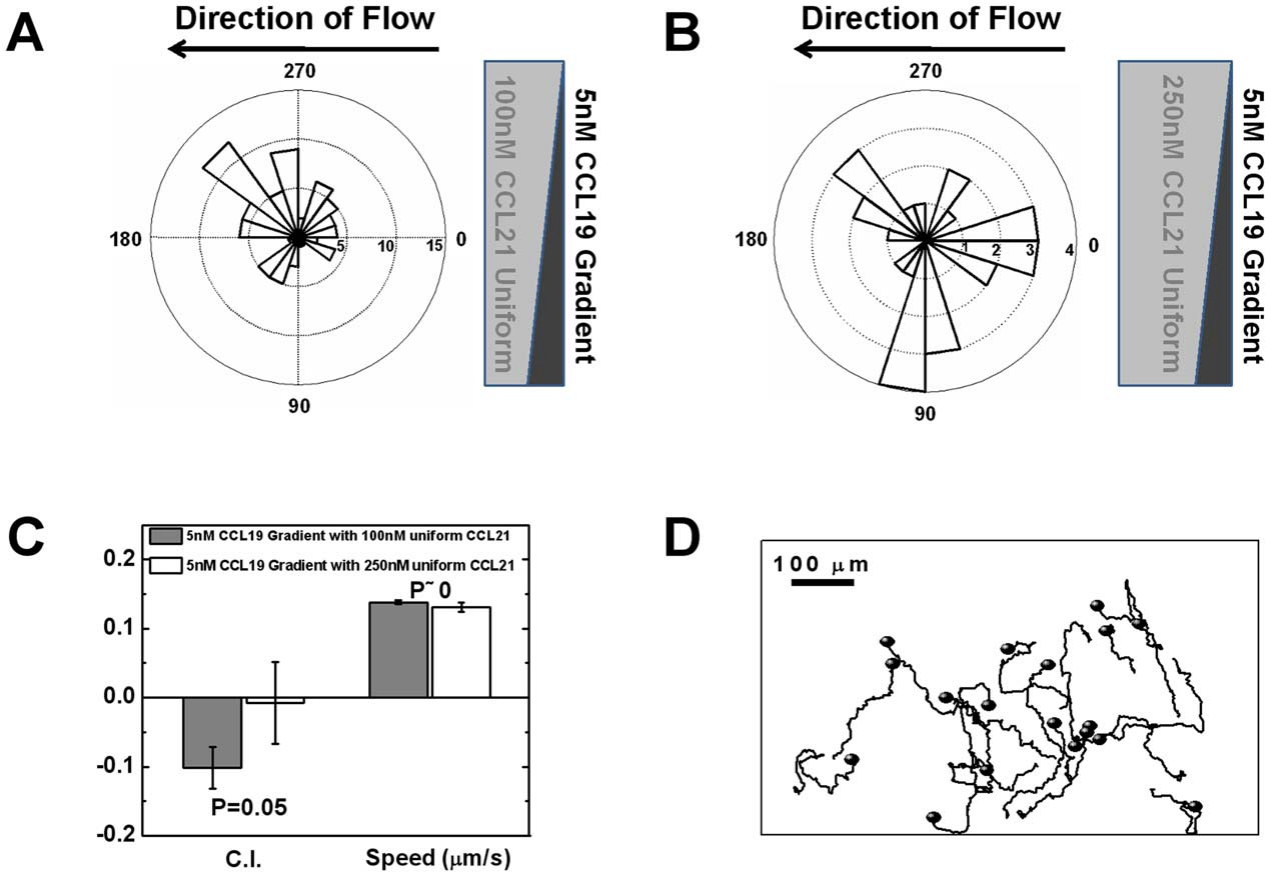
T cell migration in a CCL19 gradient with a uniform background of CCL21. (**A**) Angular histogram shows more T cells orient against a 5 nM CCL19 gradient with a uniform background of 100 nM CCL21. (**B**) Angular histogram shows T cells orient randomly in a 5 nM CCL19 gradient with a super-physiological 250 nM uniform CCL21 field. (**C**) Comparison of chemotactic index (C.I.) and the speed of cells between 5 nM CCL19 gradient with a uniform background of 100 nM CCL21 and 5 nM CCL19 gradient with a super-physiological 250 nM uniform CCL21 field. The error bars represent the standard error of the mean (s.e.m.). Positive C.I. indicates cells migrate toward the gradients. (**D**) Selected cell tracks from a representative experiment showing more cells migrate away from the 5 nM CCL19 gradient in a uniform background of 100 nM CCL21.

As discussed in the Introduction section, because of more CCL21 producing cells than CCL19 producing cells in the periphery of TCZ [21], we speculate that the chemokine field in this region to be the superposition of a CCL19 gradient and a uniform CCL21 field. Therefore, we next tested T cell migration in this gradient configuration (i.e. a 5 nM CCL19 gradient with a uniform background of 100 nM CCL21) using microfluidic devices. Unexpectedly, more T cells oriented and migrated away from the 5 nM CCL19 gradient (Figure 5A and 5D and Video S6) and this repulsive effect is shown by the relatively high negative chemotactic index, the migration angle distribution and individual cell tracks. The speed of these cells is similar to it in other chemokine fields tested in this paper. If a super-physiological concentration (i.e. 250 nM) of uniform CCL21 field is used, cells will not migrate away from the 5 nM CCL19 gradient, but migrate randomly (Figure 5B, Video S7). Additionally, we tested the condition of superimposed gradients 5 nM CCL19 and 100 nM CCL21 along the same side, and analyzed cell migration in different regions of the gradient fields. As detailed in the Supporting Information S1, our results show that in the high concentration region of the CCL19 and CCL21 gradients, cells exhibit repulsive migration away from the gradients; In contrast, in the low concentration region of the CCL19 and CCL21 gradients, cells chemotax to the gradients; in the middle region of the gradient fields, cells migrate randomly. This experiment demonstrates the differential cell migratory behaviours in different combinations of CCL19 and CCL21 fields in a single experimental setup. Taking together, these results suggest that CCL19 and CCL21 may play an interesting role together in regulating T cell migration in the periphery of TCZ.

### Differential CCR7 desensitization by CCL19 and CCL21 as an underlying mechanism

**Figure 6:**
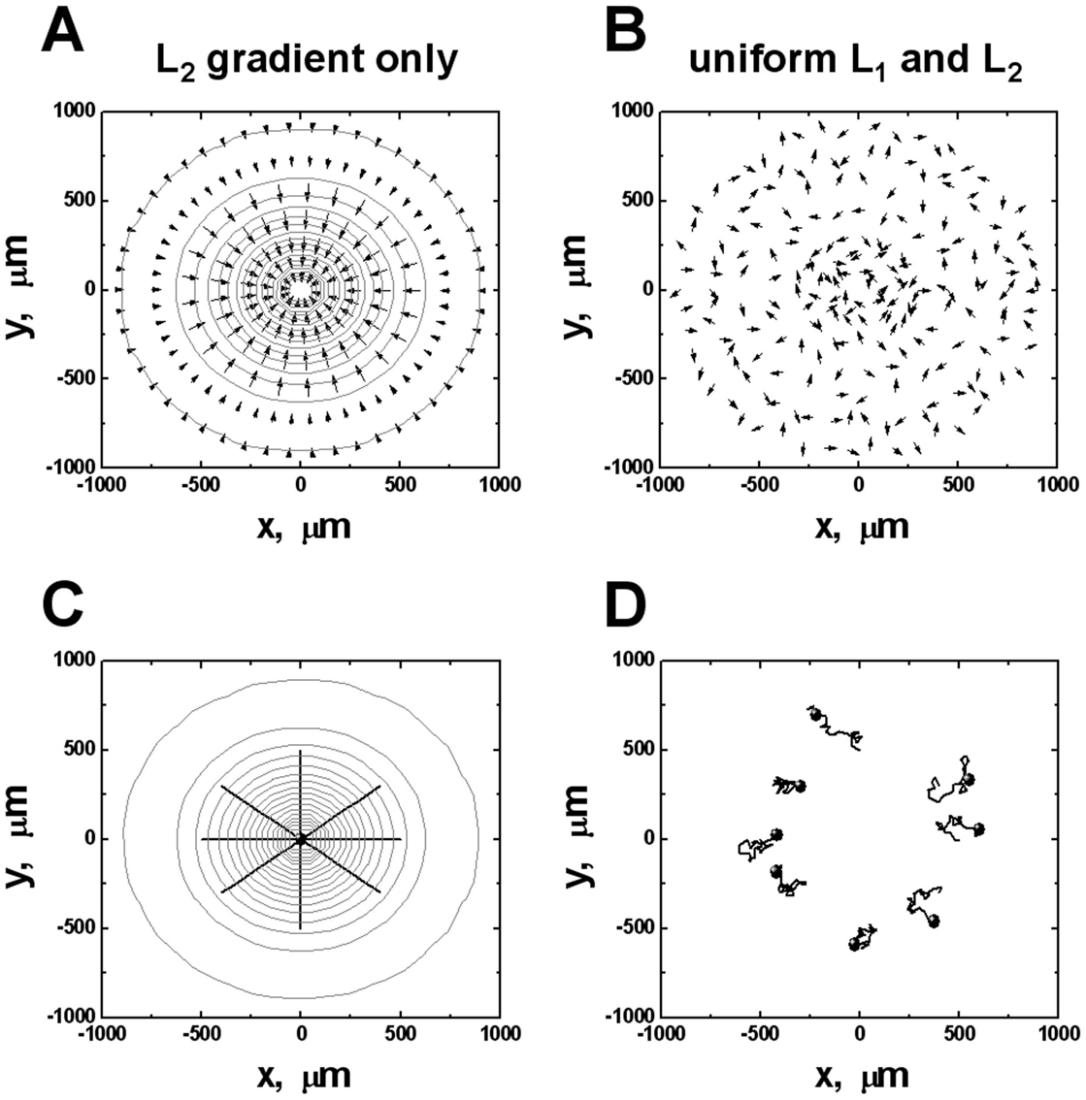
Model predictions of cell orientation and migration in a single L_2_ gradient or a “double-uniform” L_1_ and L_2_ fields. Orientation and migration of cells in a single L_2_ gradient (**A, C**) and in co-existing uniform L_1_ (0.88 nM) and L_2_ (17.6 nM) fields (**B, D**). The 20-fold concentration difference between L_1_ and L_2_ based on neutrophil parameters [28] simulates the scenario of CCL19 and CCL21 production in LNs. The cell orientation at steady state is represented by arrows in the figures and the length of the arrows indicates the strength of the orientation. The ligand gradient is represented by contour plot with the highest ligand concentration (17.6 nM) at the center of the contours for the gradient. The ligand concentration at the outmost contour circle is 0.03 nM, and the concentration difference between adjacent circles is 0.9 nM. Because of the magnitude difference between the orientation vector of the cell in different conditions, the length of the arrow is adjusted with a scaling factor of 0.07 for (A) and 15 for (B). The total time of cell migration in (C) and (D) is 150 minutes. Eight representative cell tracks are shown, and the starting positions of the tracks are consistent in all simulations. The end of the tracks is indicated by solid circles.

**Figure 7:**
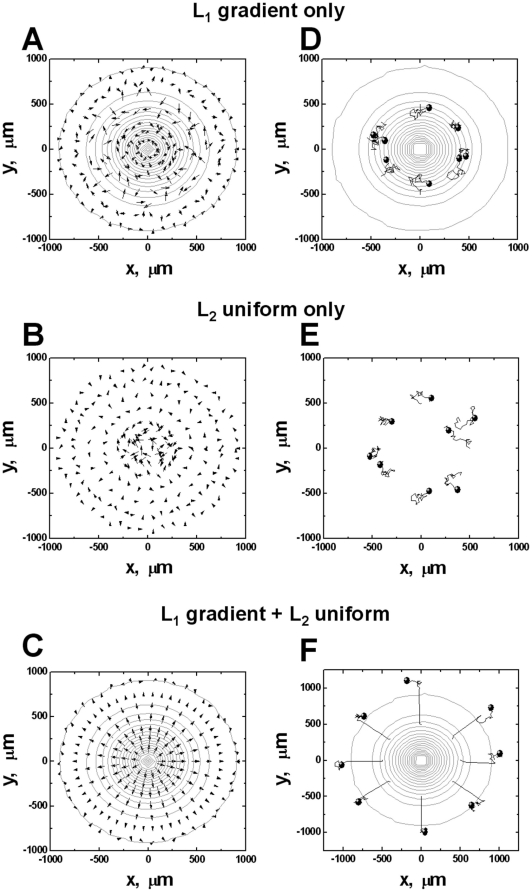
Model predictions of cell orientation and migration in a single L_1_ gradient, a uniform L_2_ field, and a single L_1_ gradient with a uniform background of L_2_. Comparison of orientation and migration of cells in single L_1_ gradient (**A, D**), in a uniform L_2_ field (**B, E**), and in co-existing L_1_ gradient and uniform L_2_ fields (**C, F**). The 20-fold concentration difference between L_1_ and L_2_ based on neutrophil parameters [28] simulates the scenario of CCL19 and CCL21 production in LNs. The cell orientation at steady state is represented by arrows in the figures and the length of the arrows indicates the strength of the orientation. The ligand gradient is represented by contour plot with the highest ligand concentration (0.88 nM) at the center of the contours for each gradient. The ligand concentration at the outmost contour circle is 0.001 nM, and the concentration difference between adjacent circles is 0.044 nM. Because of the magnitude difference between the orientation vector of the cell in different conditions, the length of the arrow is adjusted with a scaling factor of 2.25 for (A), 3302 for (B) and 0.12 for (C). Simulation results show that cells migrate randomly in a low dose single L_1_ gradient (D); In a high dose uniform L_2_ field, cells migrate randomly as expected (E); In co-existing fields of a low dose L_1_ gradient and a high dose uniform L_2_, cells migrate away from the L_1_ gradient (F). The total time of cell migration is 150 minutes. Eight representative cell tracks are shown, and the starting positions of the tracks are consistent in all simulations. The end of the tracks is indicated by solid circles.

**Figure 8:**
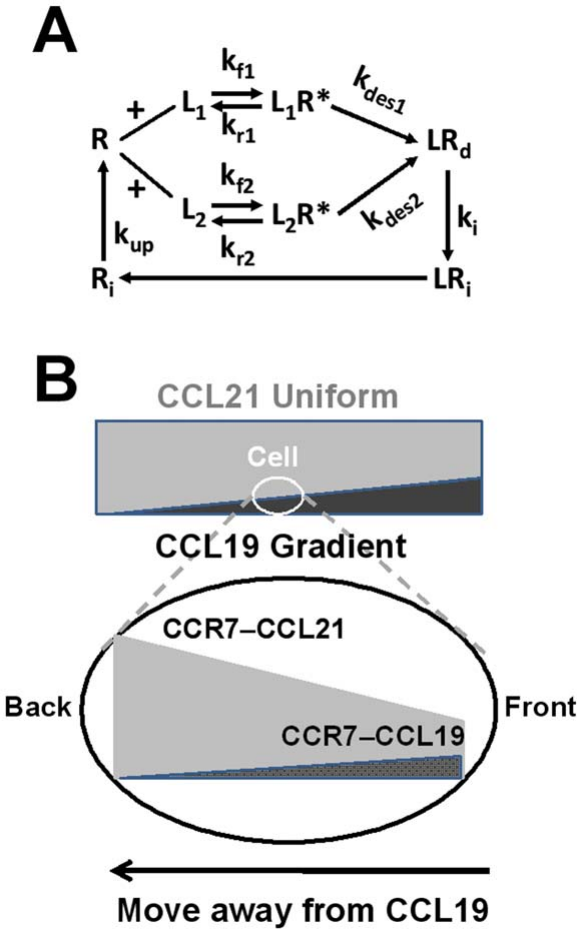
Illustration of the model and its explanation for the repulsive T cell migration. (**A**) Illustration of the model for receptor modulations by the 2 ligand L_1_ and L_2_. (**B**) The model provides an explanation for the repulsive migration of cells in a low dose desensitizing ligand gradient (L_1_ in the model and CCL19 in the experiment) with a high dose uniform background of a nondesensitizing ligand (L_2_ in the model and CCL21 in the experiment). The desensitizing ligand gradient (L_1_ in the model and CCL19 in the experiment) causes a differential receptor binding and activation between the front and the back of the cell with more activated receptors that does not lead to chemotaxis toward the gradient at low ligand dose. Although the difference of receptor activation across the cell does not lead to chemotaxis toward the gradient at the low ligand dose, it causes a difference of available free receptors between the front and the back of the cell with less free receptors in the front. As a result, when a nondesensitizing uniform ligand field (L_2_ in the model and CCL21 in the experiment) is superimposed to the desensitizing ligand gradient, the high dose nondesensitizing ligand binds and activates more receptors in the back than the front of the cell. Additionally, the nondesensitizing ligand activated receptors stay active on the cell surface for chemotactic signaling that reverses the difference of activated receptors between the front and the back of the cells with more activated receptors in the back facing the low concentration side of the desensitizing gradient. Thus, the model suggests that the differential ability of CCL19 and CCL21 for desensitizing CCR7 combined with the physiologically relevant configuration of superimposed CCL19 and CCL21 fields (possibly in the periphery of TCZ) enable the repulsive migration of T cells.

To further understand the observed repulsive migration of cells from the CCL19 gradient in the uniform background of CCL21, we adapted a previous mathematical model to consider the ligand-induced chemoattractant receptor modulations for mediating cell orientation and migration in ligand fields [28]. The previous model is modified to consider 2 ligands L_1_ and L_2_ with a common cell receptor R. We assume only L_1_ but not L_2_ can desensitize R to simulate the differential ability of CCL19 and CCL21 for desensitizing CCR7. In a high dose L_2_ gradient, the model predicts that cells orient and migrate toward the L_2_ gradient (Figure 6A, 6C and Video S8). In a superimposed field of low dose uniform L_1_ and high dose uniform L_2_, the model predicts that cells orient and migrate randomly (Figure 6B, 6D and Video S11). These predictions are in agreement with our experimental results of T cell migration in single 100 nM CCL21 gradient (Figure 2 and Video S1) and in “double-uniform” CCL19 and CCL21 fields (Figure 4 and Video S5). Furthermore, the model predicts that cells orient and migrate randomly in a low dose L_1_ gradient (Figure 7A, 7D and Video S10) or a high dose uniform field of L_2_ (Figure 7B, 7E and Video S9), consistent with experimental results of random migration of T cells in a 5 nM CCL19 gradient or a 100 nM uniform CCL21 field (Figure 3 and Figure 2; Videos S3 and S2). In the configuration of a low dose L_1_ gradient with a uniform background of high dose L_2_, the model predicts the repulsive migration of cells from the L_1_ gradient (Figure 7C and 7F and Video S12) and this prediction is in consistency with the experimentally observed repulsive migration of T cells from the 5 nM CCL19 gradient in a uniform background of 100 nM CCL21 (Figure 5 and Video S6). Thus, the modeling predictions and experimental results are in good agreement. The model is illustrated in Figure 8A with more details in the Materials and Methods section and the Supporting Information S1.

As illustrated in Figure 8B, mathematical modeling provides an explanation for the repulsive migration of cells in a low dose desensitizing ligand gradient (L_1_ in the model and CCL19 in the experiment) with a high dose uniform background of a nondesensitizing ligand (L_2_ in the model and CCL21 in the experiment). The desensitizing ligand gradient (L_1_ in the model and CCL19 in the experiment) causes a differential receptor binding and activation between the front and the back of the cell with more activated receptors in the front. Although the difference of receptor activation across the cell does not lead to chemotaxis toward the gradient at the low ligand dose, it causes a difference of available free receptors between the front and the back of the cell with less free receptors in the front. As a result, when a nondesensitizing uniform ligand field (L_2_ in the model and CCL21 in the experiment) is superimposed to the desensitizing ligand gradient, the high dose nondesensitizing ligands bind and activate more receptors in the back than the front of the cell. Additionally, the nondesensitizing ligand activated receptors stay active on the cell surface for chemotactic signaling that reverses the difference of activated receptors between the front and the back of the cells with more activated receptors in the back facing the low concentration side of the desensitizing gradient. Thus, the model suggests that the differential ability of CCL19 and CCL21 for desensitizing CCR7 combined with the hypothesized physiological configuration of superimposed CCL19 and CCL21 fields (possibly in the periphery of TCZ) enable the repulsive migration of T cells.

### Hypothesized combinatorial guidance by CCR7 ligands for T cell migration

**Figure 9:**
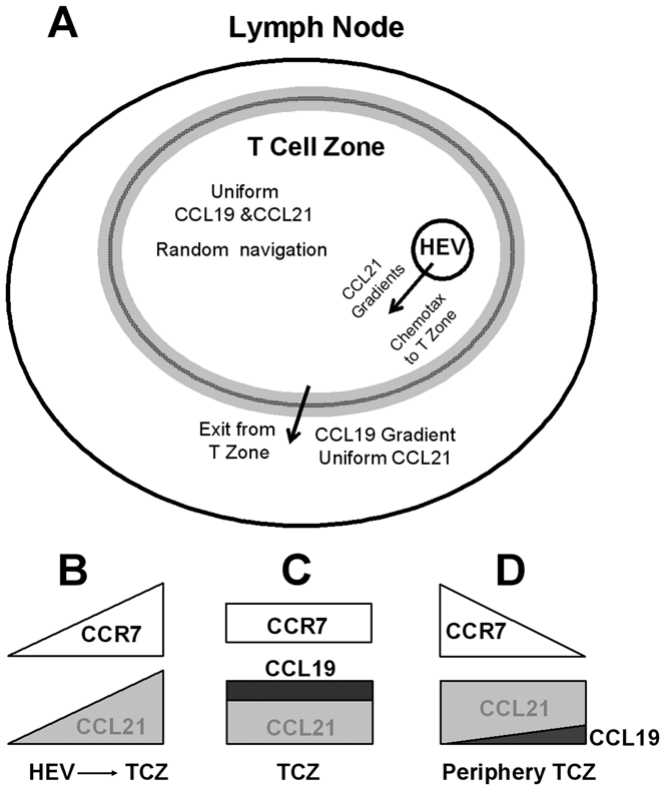
The proposed combinatorial guidance mechanism. (**A**) We propose a possible combinatorial guiding mechanism by different configurations of CCL19 and CCL21 gradient fields for T cell migration in different sub-regions of lymph nodes. CCL21 alone mediates the entry of T cells to the TCZ of LNs through HEV. Inside TCZ, T cells migrate randomly in uniform fields of CCL19 and CCL21 to maximize sampling efficiency with antigen presenting cells (APCs) for immune synaptic interactions. The exit of T cells from LNs is facilitated by first migrating out of TCZ through a CCR7-dependent mechanism. Specifically, T cells migrate away from TCZ when they reach (by random migration) the periphery region of TCZ wherein the gradient fields is likely to be a superposition of a low dose CCL19 gradient and a high dose uniform CCL21 field. This mechanism is enabled by the competition of CCR7 binding between CCL19 and CCL21, together with the differential ability of CCL19 and CCL21 for desensitizing CCR7 and the unique superimposed chemokine field profiles. Such combinatorial guiding mechanism argues the importance and necessity of co-expression of CCL19 and CCL21 in TCZ and the robust design for T cell entry to LNs, navigation within LNs, and exit from LNs using a united CCR7-dependent mechanism. (**B–D**) Schematic illustration of the hypothesized chemokine fields in different regions in the lymph nodes and the corresponding distributions of signalling CCR7 on the cell surface indicating the cell orientation and migration direction.

Taking together our experimental and modeling results and the previous results of others, we propose a possible combinatorial guiding mechanism by different configurations of CCL19 and CCL21 gradient fields for T cell migration in different sub-regions of LNs (Figure 9): Although both CCL19 and CCL21 are chemoattractants for T cells, CCL21 alone is sufficient to mediate the entry of T cells to the TCZ of LNs through HEV. This is supported by our results showing T cells chemotax to a 100 nM CCL21 gradient (Figure 2 and Video S1) but not a 5 nM CCL19 gradient (Figure 3 and Video S3) as well as by previous experimental studies [20], [25]. Inside TCZ, T cells migrate randomly in uniform fields of CCL19 and CCL21 to maximize sampling efficiency with antigen presenting cells (APCs) for immune synaptic interactions [29], [30], [31]. As expected, our results show random migration of T cells in “double-uniform” CCL19 and CCL21 fields (Figure 4 and Video S5). Thus, CCR7 and CCL21 play important roles in T cell recruitment to LNs, and in T cell migration within TCZ. However, CCL19 is not necessarily required for these processes. Previous studies suggested that CCR7 down-regulation combined with S1P signaling mediate the exit of T cells from LNs for recirculation and immune responses [11], [32]. The exit process will be facilitated if T cells first migrate out of TCZ through a CCR7-dependent mechanism.

Here we suggest that T cells will migrate away from TCZ when they reach (by random migration) the periphery region of TCZ wherein the gradient fields is speculated to be a superposition of a low dose CCL19 gradient and a high dose uniform CCL21 field. This mechanism is enabled by the competition of CCR7 binding between CCL19 and CCL21, together with the differential ability of CCL19 and CCL21 for desensitizing CCR7 and the unique superimposed chemokine field profiles. Interestingly, this CCR7-dependent mechanism for T cell exit from SLT responds well to the previously reported CCR7-dependent T cell exit from peripheral tissues [27], [33], suggesting the importance of CCR7 in T cell trafficking and recirculation. Furthermore, it has been previously reported that leukocytes exhibit repulsive migration from high concentration chemoattractant gradients, termed “chemofugetaxis” [34], [35], [36], [37] and that receptor desensitization may play a role in such chemorepulsive migration [38]. Thus, there are other mechanisms for the repulsive cell migration that differ from the proposed mechanism of this study based on the combined chemokine fields. Altogether, this combinatorial guiding mechanism argues for the importance and necessity of co-expression of CCL19 and CCL21 in TCZ and the robust design for T cell entry to LNs, navigation within LNs, and exit from LNs using a united CCR7-dependent mechanism in combination with other important mechanisms such as S1P signalling.

The differential ability of CCL19 and CCL21 for desensitizing CCR7 has been demonstrated previously [24]. In addition, the physiological concentrations of CCL19 and CCL21 in LNs were approximated according to previous studies [21]. It is technically challenging to quantitatively measure the gradient profiles of CCL19 and CCL21 *in vivo*, especially considering the low amount of CCL19 in LNs, and so far there is no published data available. In addition, it is difficult to test cell migration in complex co-existing chemokine fields using conventional assays such as transwell assays. The microfluidic devices used this study allowed us to quantitatively test T cell migration in different chemokine gradient conditions that mimic possible scenarios in LNs. The results of such studies in conjunction with mathematical modeling and computer simulations offer novel insights into the complex process of T cell migration and trafficking in SLT.

In summary, we experimentally investigated T cell migration in different single and superimposed CCL19 and CCL21 fields using microfluidic devices. Our results show that the CCL21 gradient but not the CCL19 gradient at physiological concentrations similar to those observed in LNs attract T cells *in vitro*. T cells migrate randomly in “double-uniform” fields of CCL19 and CCL21. However, T cells migrate away from the CCL19 gradient in the presence of a uniform background of CCL21. The experimental results are consistent with mathematical modeling and computer simulations, and the repulsive migration of T cells is explained by mathematical modeling based on the chemokine field profiles, competition of CCL19 and CCL21 for activating CCR7 and the differential ability of CCL19 and CCL21 for desensitizing CCR7. Based on these results, we propose a combinatorial guiding mechanism by CCL19 and CCL21 for T cell migration in LNs.

## Materials and Methods

### T cell preparation

Human peripheral blood samples were collected from healthy donors in collaboration with The Victoria General Hospital at Winnipeg with an approved human ethics protocol. Peripheral blood mononuclear cells (PBMC) were isolated using standard gradient centrifugation method. T cells from total PBMC were selectively activated by anti-CD3/CD28 antibodies for 2 days in culture medium (RPMI-1640 with 1% PS and 10% FBS) in a 37°C incubator with 8% CO_2_ injection. Activated T cells were expanded with IL-2 and were cultured for at least 3 days before cell migration experiments.

### Microfluidic device and gradient generation

A previously reported “Y” shape microfluidic device was used for cell migration experiments in this study (Figure 1) [39]. The microfluidic device was designed in Freehand 9.0 (Macromedia) and the design was printed to a transparency mask by a high resolution printer. The masters were fabricated at Stanford Nanofabrication Facility (SNF) at Stanford University and The Nano Systems Fabrication Laboratory (NSFL) at the University of Manitoba. The design was patterned on a silicon wafer by contact photolithography with SU-8 photoresist (Micro Chem, MA) through the transparency mask and the SU-8 pattern yields ∼100 µm thickness. Two 1 mm diameter holes for the 2 fluidic inlets and one 4 mm diameter hole for the fluidic outlet were punched out of PDMS respectively in the device. An additional 1 mm hole was punched for loading cells. The PDMS replicas were then fabricated by molding PDMS (Sylgard 184 silicon elastomer, Dow Corning, MI) against the master, and were bonded to a glass slide using an air plasma cleaner. Polyethylene tubing (PE-20, Becton Dickinson, MD) was inserted into the inlet holes to connect the microfluidic device to syringe pumps (Model V6, Kloehn, Inc., NV) with two 250 µL Kloehn syringes containing medium or chemokine solutions for fluidic infusion. Chemokine solutions (Recombinant Human CCL19/MIP-3 beta and Recombinant Human CCL21/6Ckine from R&D Systems) of suitable concentrations were prepared in migration medium (RPMI-1640 with 0.4% BSA). FITC-Dextran 10 kD that has similar molecular weight of the chemokine molecule was added to the chemokine solution. The migration medium and chemokine solutions were continuously infused into the device by syringe pumps through tubing and the inlets of the device at the total flow rate of 0.2 µL/min. The defined stable chemokine gradients are generated by controlled mixing of chemokines and medium. The chemokine gradient was confirmed by measuring the fluorescence intensity profile of FITC-Dextran inside the microfluidic channel and the cells were imaged at ∼3 mm downstream of the “Y” junction where the gradient yields a smooth profile. For generating superimposed CCL19 and CCL21 fields, solutions of one or both chemokines with specific concentrations were used for both inlets (i.e. CCL19 and CCL21 were infused to both inlets for “double-uniform” fields; CCL19 was infused to one inlet and CCL21 were infused to both inlets for generating a CCL19 gradient with a uniform background of CCL21).

### Cell migration experiments

The fluidic channel was coated with fibronectin (BD Biosciences, MA) for 1 hour at room temperature and blocked with BSA for another hour before the experiment. For each experiment, cells were loaded into the microfluidic device from the wells and allowed to settle in the fibronectin-coated channel for ∼5 min. The device was maintained at 37°C by attaching a transparent heater to the back of the cover slide (Thermal-Clear Transparent Heater, Model No. H15227, Minco, MN). The heater was powered by a DC power supply (Model No. 6204A, Harrison, Canada) and was controlled by a sensorless temperature controller (Model No. CT198, Minco, MN). The temperature was calibrated to 37°C using a digital thermometer (VWR, Canada). Medium and chemokine solutions were infused into the device by syringe pumps through tubing and the inlets of the device. The device was placed on a microscope stage (Model No. BX60, Olympus). The system was allowed to equilibrate for ∼5 min (wait until no flowing cells were seen in the channel) and cell migration was recorded by time-lapse microscopy at 6 frames/min for 19 to 44 min using a CCD camera (Model No. 370 KL 1044, Optikon, Canada). The image acquisition was controlled by NIH ImageJ (v.1.34s).

### Data analysis

Movement of individual cells was tracked using NIH ImageJ (v.1.34s). The background noise of the image was removed using the “despeckle” function. Then the images were calibrated to distance. Only the cells that migrated within the microscope field were selected and tracked using the “Manual Tracking” plug-in in NIH ImageJ. The tracking data were exported to Excel and MATLAB for analysis. Following previously established analysis methods [39], [40], the movement of cells was quantitatively evaluated by (a) the percentage of cells that migrated toward the chemokine gradient; (b) the Chemotactic Index (C.I.), which is the ratio of the displacement of cells toward the chemokine gradient (Δ*y*), to the total migration distance (*d*) using the equation C.I. = *Δy*/*d*, presented as the average value ± standard error of the mean (s.e.m); (c) the average speed (*V*) calculated as *d*/Δ*t* and presented as the average value ± s.e.m. of all cells; and (d) statistical analysis of migration angles performed using MATLAB to examine the directionality of the cell movement. Specifically, migration angles (calculated from *x*-*y* coordinates at the beginning and the end of the cell tracks) were summarized in a direction plot, which is a rose diagram showing the distribution of angles grouped in defined intervals, with the radius of each wedge indicating cell number. The parameters between different conditions were compared by the 2 sample *t* test. 20–85 cells were analyzed for each experiment. Two-three independent experiments were repeated for each condition with similar results. The figures in the paper were generated using one representative experiment for each condition.

### Mathematical modeling and computer simulations

A previous cell gradient sensing model was adapted to describe receptor-ligand binding, receptor desensitization and recycling [28]. As illustrated in Fig. 8A, two ligands L_1_ and L_2_ share a common cell receptor R with equal binding affinity. However, only L_1_ but not L_2_ desensitizes R. Desensitized receptors are subsequently internalized and eventually re-expressed back to the cell surface. The model cell is simplified to consist of four receptor expressing units symmetrically located along the x and y axis with equal distance to the center of mass of the cell (r = 5 µm assuming the typical 10 µm diameter of cells [41]. A set of ordinary differential equations (ODEs) are used to describe the evolution of ligand-induced receptors modulations. In a single ligand field, the active receptor-ligand complex LR* is evaluated for all four receptor expressing units of the cell, and the difference of LR* along the x and y axis is calculated to determine the orientation strength in the two directions. The net orientation of the cell is determined by the orientation vector 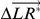 in the 2-D plane. In superimposed ligand fields of L_1_ and L_2_, the net orientation vector of the cell is determined by the addition of the orientation vectors to L_1_ and L_2_: 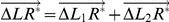. The threshold magnitude of the orientation vector for chemotactic orientation is set at 10, i.e. |ΔLR*|≥10. Below the threshold, i.e. |ΔLR*|<10, the cell orients and migrates randomly in the 2-D plane [28], [42], [43]. The cell orientation at long time is determined by evaluating 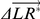 at the equilibrium state (i.e. 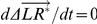). Based on the gradient sensing model, the migration of the model cell is simulated by continuously evaluating the orientation vector and allowing the cell to move along the direction set by the net orientation vector. More details of the model, and the parameters and their values used in the model are provided in the Supporting Information S1.

## Supporting Information

Supporting Information S1 **Supporting methods for mathematical modeling and computer simulations; Supporting results on cell migration in same side CCL19 and CCL21 gradients; Supporting table, figure, and references.**
(DOC)

Video S1 **Chemotaxis of T cells in a 100 nM CCL21 gradient.**
(MOV)

Video S2 **Random migration of T cells in a uniform 100 nM CCL21 field.**
(MOV)

Video S3 **Random migration of T cells in a 5 nM CCL19 gradient.**
(MOV)

Video S4 **Chemotaxis of T cells in a 100 nM CCL19 gradient.**
(MOV)

Video S5 **Random migration of T cells in “double-uniform” fields of 5 nM CCL19 and 100 nM CCL21.**
(MOV)

Video S6 **Repulsive migration of T cells from a 5 nM CCL19 gradient with a 100 nM CCL21 uniform background.**
(MOV)

Video S7 **Random migration of T cells from a 5 nM CCL19 gradient with a 250 nM CCL21 uniform background.**
(MOV)

Video S8 **Simulation shows chemotaxis of cells in a high dose L_2_ gradient.**
(MOV)

Video S9 **Simulation shows random migration of cells in a uniform L_2_ field.**
(MOV)

Video S10 **Simulation shows random migration of cells in a low dose L_1_ gradient.**
(MOV)

Video S11 **Simulation shows random migration of cells in “double-uniform” fields of low dose L_1_ and high dose L_2_.**
(MOV)

Video S12 **Simulation shows repulsive migration of cells from a low dose L_1_ gradient with a high dose L_2_ uniform background.**
(MOV)
