# Supplementary material for: Combinatorial Guidance by CCR7 Ligands for T Lymphocytes Migration in Co-Existing Chemokine Fields
Source: PLoS One. 2011 Mar 25;6(3):e18183. doi: 10.1371/journal.pone.0018183 (PMC3064588; doi:10.1371/journal.pone.0018183)
Supplement: Supporting Information S1 — Supporting methods for mathematical modeling and computer simulations; Supporting results on cell migration in same side CCL19 and CCL21 gradients; Supporting table, figure, and references. (DOC) [file pone.0018183.s001.doc]

**Combinatorial Guidance by CCR7 Ligands for T Lymphocytes Migration in Co-Existing Chemokine Fields**

Saravanan Nandagopal, Dan Wu, and Francis Lin

**Supporting Information S1**

1. **Mathematical modeling and computer simulations**

A previous cell gradient sensing model was adapted to describe receptor-ligand binding, receptor desensitization and recycling [1]. As illustrated in Fig. 8A, receptors are initially expressed on the cell surface. Upon binding to the ligand molecules, the receptors are activated and the activated receptors trigger chemotactic signaling. Two ligands L_1_ and L_2_ share a common cell receptor R with equal binding affinity. However, only L_1_ but not L_2_ desensitizes R. Desensitized receptors are subsequently internalized and eventually re-expressed back to the cell surface. Consistent with previous models, the dissociation of ligand from desensitized receptors on the cell surface is assumed negligible [2]. The model cell is simplified to consist of four receptor expressing units symmetrically located along the x and y axis with equal distance to the center of mass of the cell (r=5μm assuming the typical 10μm diameter of cells [3].

A set of ordinary differential equations (ODEs) are used to describe the evolution of ligand-induced receptors modulations. The symbols for variables and kinetic rates in the model are defined in Table S1 and the values of kinetic rates and other parameters are adapted from the literature based on human neutrophil formyl peptide receptors [1,2,4,5]. Because of the similarity of neutrophils and T cells in their size, the mechanisms and characteristics for gradient sensing and migration and that the specific kinetic parameters for T cells are not available in existing literature, here we setup the model and simulations based on neutrophil parameters.

(1)

$\frac{dL_{2}R^{*}}{dt}=k_{f2}\times L_{2}\times R-k_{r2}\times L_{2}R^{*}-k_{des2}\times L_{2}R^{*}$ (2)

$\frac{dLR_{d}}{dt}=k_{des1}\times L_{1}R^{*}+k_{des2}\times L_{2}R^{*}-k_{i}\times LR_{d}$ (3)

$\frac{dR}{dt}=k_{r1}\times L_{1}R^{*}+k_{r2}\times L_{2}R^{*}-k_{f1}\times L_{1}\times R-k_{f2}\times L_{2}\times R+k_{up}\times R_{i}$ (4)

The ODEs are subject to restraint conditions assuming total receptor conservation and that all receptors are initially expressed on the cell surface in the free & nondesensitized receptor state.

$R_{tot}=R+R_{i}+L_{1}R^{*}+L_{2}R^{*}+LR_{d}= cons$ (5)

$R_{t=0}=R_{tot}$ (6)

$R_{i,t=0}=0$ (7)

(8)

$L_{2}R^{*}_{t=0}=0$ (9)

$LR_{d,t=0}=0$ (10)

Fixed nonlinear ligand gradients in a 2-D polar coordinate system are set up in the model. The parameters are defined in Table S1 and the selection of this gradient has been justified previously [1].

$L=\frac{L_{\max}}{A^{n}}\times(A-\rho)^{n}+L_{0}$ (11)

In a single ligand field, the active receptor-ligand complex LR* is evaluated for all four receptor expressing units of the cell, and the difference of LR* along the x and y axis is calculated to determine the orientation strength in the two directions. The net orientation of the cell is determined by the orientation vector *\s\up5(→)* in the 2-D plane.

$\overrightarrow{\Delta L_{1}R^{*}}=(L_{1}R_{x+r}^{*}-L_{1}R_{x-r}^{*})\hat{i}+(L_{1}R_{y+r}^{*}-L_{1}R_{y-r}^{*})\hat{j}$ (12)

$\overrightarrow{\Delta L_{2}R^{*}}=(L_{2}R_{x+r}^{*}-L_{2}R_{x-r}^{*})\hat{i}+(L_{2}R_{y+r}^{*}-L_{2}R_{y-r}^{*})\hat{j}$ (13)

In superimposed ligand fields of L_1_ and L_2_, The net orientation vector of the cell is determined by the addition of the orientation vectors to L_1_ and L_2_.

$\overrightarrow{\Delta LR^{*}}=\overrightarrow{\Delta L_{1}R^{*}}+\overrightarrow{\Delta L_{2}R^{*}}$ (14)

The differential equations are integrated by the 4th order Runge-Kutta algorithm. Consistent with the previous models [1,6,7], the threshold magnitude of the orientation vector for chemotactic orientation is set at 10, i.e. *|ΔLR*|* ≥ 10. Below the threshold, i.e. *|ΔLR*|* <10, the cell orients and migrates randomly in the 2-D plane. The random orientation in the 2-D plane (0, 2π) is determined by a random number generator. The cell orientation at long time is determined by evaluating *\s\up5(→)* at the equilibrium state (i.e. *d\s\up5(→)* */dt=0*).

Based on the gradient sensing model, the model cell is allowed to move along the direction set by the net orientation vector. Initially, cells are located at different positions in the gradient fields to start migrating. The gradient sensing model as described above is applied to cells to determine their net orientation vector. The migration step is set at 2.5 minutes at 10 μm/min similar to the previously reported characteristic time of persistent cell migration [7] and migration speed [8]. The orientation vector is continuously evaluated at time step of 10 seconds and determines the direction of cell migration at each migration step.

1. **Cell migration in same side CCL19 and CCL21 gradients**

We tested the condition of superimposed gradients of 5nM CCL19 and 100nM CCL21 along the same side, and analyzed cell migration in different regions of the gradient fields (Fig. S1). Our results show that in the high concentration region of the CCL19 and CCL21 gradients cells exhibit repulsive migration away from the gradients; In contrast, in the low concentration region of the CCL19 and CCL21 gradients cells chemotax to the gradients; in the middle region of the gradient fields, cells migrate randomly. This experiment demonstrates the differential cell migratory behaviours in different combinations of CCL19 and CCL21 fields in a single experimental setup.

**Figure S1.**


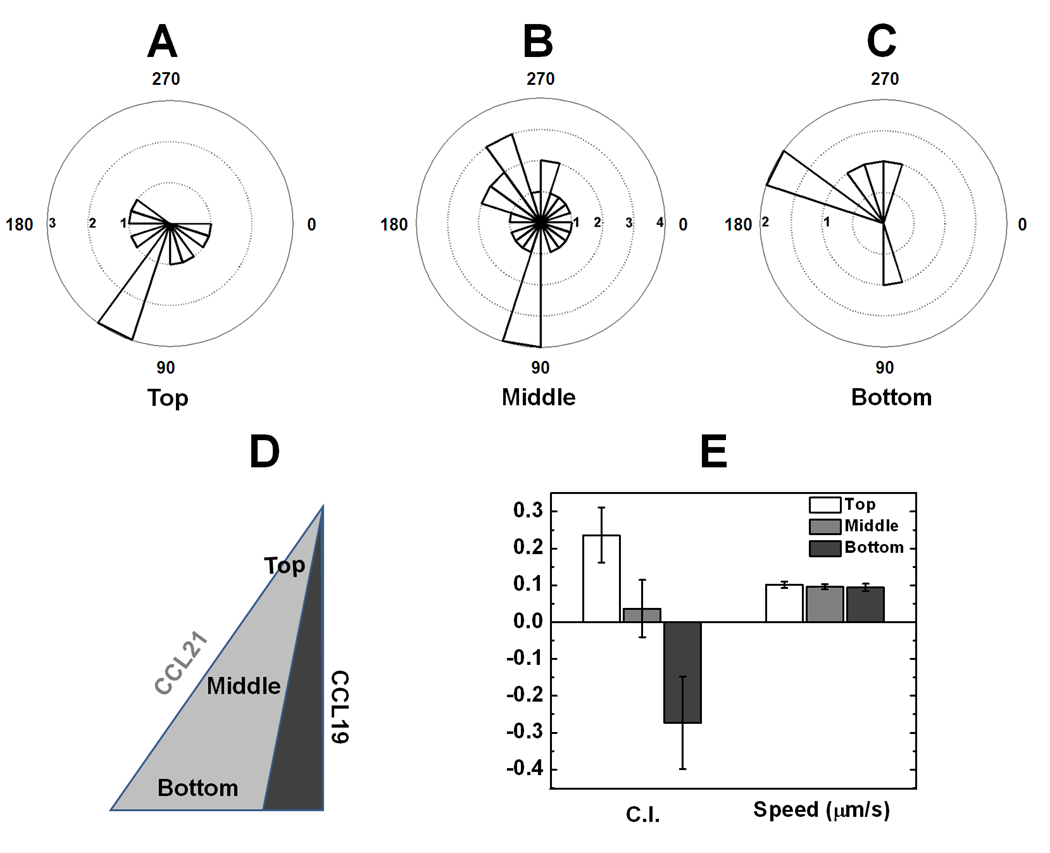


**T cell migration in same side gradient fields of 5nM CCL19 and 100nM CCL21. (A)** Angular histogram shows that T cells in the top region orient towards the CCL19/CCL21gradients. **(B)** Angular histogram shows that T cells in the middle region orient randomly. **(C)** Angular histogram shows that T cells in the bottom region orient away from the CCL19/CCL21 gradients. **(D)**
Schematic illustration of the same side gradient configuration and the sub-regions for analysis. **(E)** Comparison of chemotactic index (C.I.) and the speed of cells in different sub-regions of the same side gradient fields. The error bars represent the standard error of the mean (s.e.m.). Positive C.I. indicates cells migrate toward the gradients and negative C.I. indicates cells migrate away from the gradients.

**Table S1.**

**Variables and parameters in the model**

| **Symbols** | **Implications** | **Values** |
| --- | --- | --- |
| *L_1_, L_2_* | ligand concentration | Variable (in nM) |
| *R* | number of free surface receptors | Variable |
| *R_i_* | number of intracellular free receptors | Variable |
| *L_1_R^*^, L_2_R^*^* | number of active receptor-ligand complex | Variable |
| *LR_d_* | number of desensitized receptor-ligand complex | Variable |
| *LR_i_* | number of internalized receptor-ligand complex | Variable |
| *R_tot_* | total number of receptors | 25,000 [1] |
| *k_f1_ , k_f2_* | ligand receptor association rate for *L_1_R** and *L_2_R** | 8.4 × 10^7^ M^-1^ s^-1^ [2,9] |
| *k_r1_, k_r2_* | low-affinity ligand receptor dissociation rate for *L_1_R** and *L_2_R** | 0.37 s^-1^ [2,5] |
| *k_des1_, k_des2_* | desensitization rate for *L_1_R** and *L_2_R** | *k_des1_ =* 0.065 s^-1^ for *L_1_R** [2,5]  *k_des2_* = 0 for *L_2_R** |
| *k_i_* | internalization rate | 0.0033 s^-1^ [2,10] |
| *k_up_* | up-regulation rate | 0.004 s^-1^ [2,4] |
| *L_max_* | highest concentration at the gradient center | 0.88 nM unless stated otherwise |
| *L_0_* | basal ligand concentration | 0 nM [1] |
| *A* | radius of the gradient region | 1000 μm [1] |
| *ρ*  *n* | distance from the gradient center  power of the gradient function | Variable (in μm) [1]  3 [1] |

**references**

1. Lin F, Butcher EC (2008) Modeling the role of homologous receptor desensitization in cell gradient sensing. J Immunol 181: 8335-8343.

2. Hoffman J, Linderman J, Omann G (1996) Receptor up-regulation, internalization, and interconverting receptor states. Critical components of a quantitative description of N-formyl peptide-receptor dynamics in the neutrophil. J Biol Chem 271: 18394-18404.

3. Lauffenburger DA (1982) Influence of external concentration fluctuations on leukocyte chemotactic orientation. Cell Biophysics 4: 177-209.

4. Norgauer J, Eberle M, Fay S, Lemke H, Sklar L (1991) Kinetics of N-formyl peptide receptor up-regulation during stimulation in human neutrophils. J Immunol 146: 975-980.

5. Sklar L, Bokoch G, Button D, Smolen J (1987) Regulation of ligand-receptor dynamics by guanine nucleotides. Real-time analysis of interconverting states for the neutrophil formyl peptide receptor. J Biol Chem 262: 135-139.

6. Lauffenburger DA, Zigmond SH (1981) Chemotactic factor concentration gradients in chemotaxis assay systems. J Immunol Methods 40: 45-60.

7. Tranquillo RT, Lauffenburger DA, Zigmond SH (1988) A stochastic model for leukocyte random motility and chemotaxis based on receptor binding fluctuations. J Cell Biol 106: 303-309.

8. Moghe PV, Nelson RD, Tranquillo RT (1995) Cytokine-stimulated chemotaxis of human neutrophils in a 3-D conjoined fibrin gel assay. Journal of Immunological Methods 180: 193-211.

9. Sklar L, Finney D, Oades Z, Jesaitis A, Painter R, et al. (1984) The dynamics of ligand-receptor interactions. Real-time analyses of association, dissociation, and internalization of an N-formyl peptide and its receptors on the human neutrophil. J Biol Chem 259: 5661-5669.

10. Sklar L (1986) Ligand-receptor dynamics and signal amplification in the neutrophil. Adv Immunol 39: 95-143.
